# Supplementary figures and images for: VAL genes regulate vegetative phase change via miR156-dependent and independent mechanisms
Source: PLoS Genet. 2021 Jun 28;17(6):e1009626. doi: 10.1371/journal.pgen.1009626 (PMC8270478; doi:10.1371/journal.pgen.1009626)

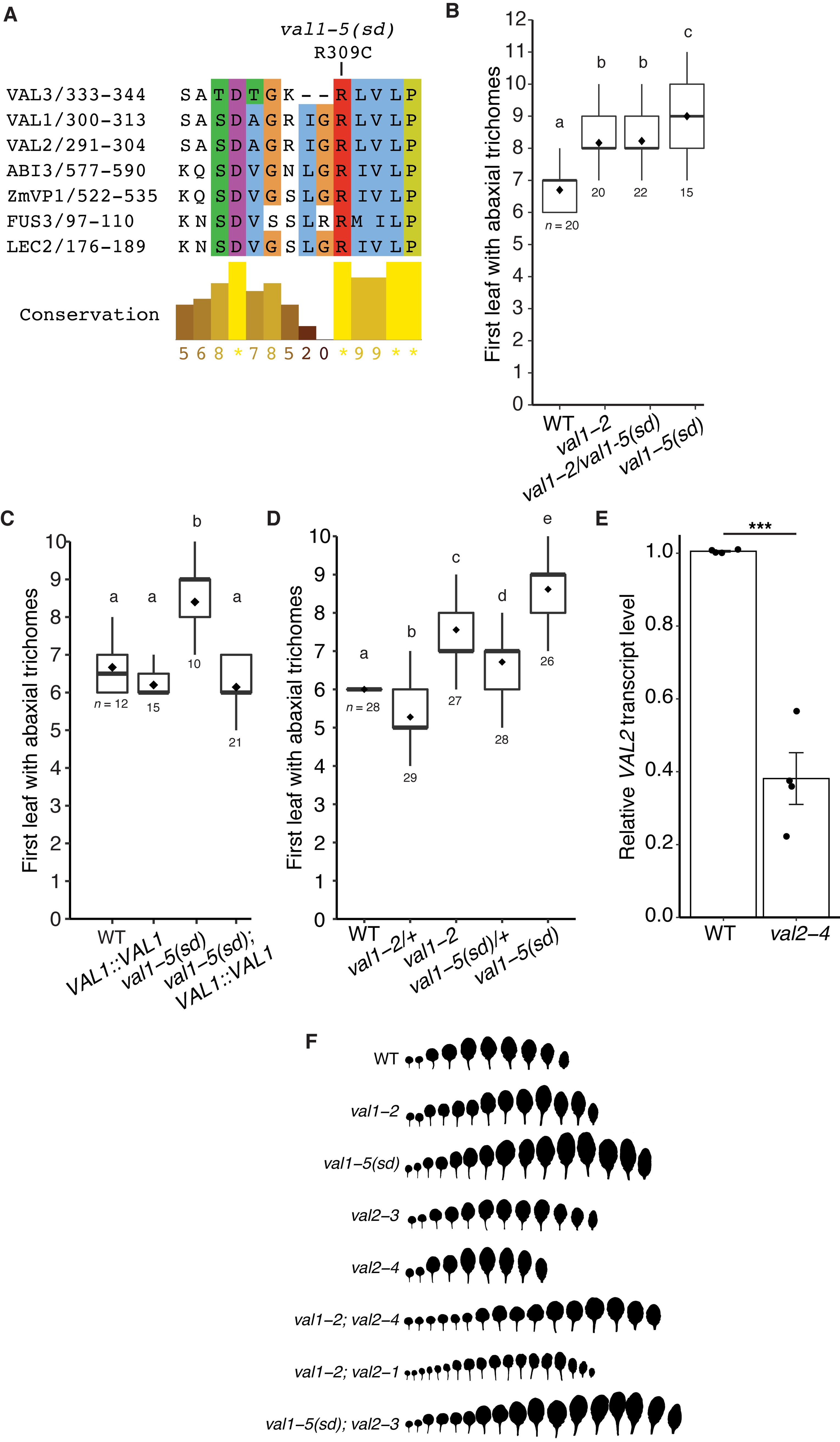

Supplement: S1 Fig — (A) Sequence alignment of the B3 DNA-binding domain N-arm of Arabidopsis LAV family members and the maize ABI3 ortholog VP1. Numbers correspond to amino acid sequence position, colors correspond to the ClustalX amino acid color scheme. In the val1-5(sd) mutant a C>T base substitution converts an arginine to a cysteine. (B) val1-5(sd) complementation test with the null val1-2 allele. (C) Rescue of the val1-5(sd) abaxial trichome phenotype with a VAL1 genomic sequence. Independent T1 lines are shown. (D) Allele heterozygosity testing. (B-D) Boxes display the interquartile range (IQR) (boxes), median (lines) and values beyond 1.5*IQR (whiskers); mean values are marked by υ. Samples sizes are displayed on the graph. Statistically distinct genotypes were identified by one-way ANOVA with post hoc Tukey multiple comparison test (letters indicate statistically distinct groups; P < 0.05), all plants grown in LD. (E) qRT-PCR analysis of gene expression in whole seedlings harvested at 7 DAG in LD conditions. Each data point represents a biological replicate and is the average of three technical replicates. Bars represent the mean and error bars mean±s.e.m. Asterisks represent significant difference between WT and val2-4 calculated by an unpaired two-tailed t-test (*** P < 0.0005). (F) Heteroblastic series of lines shown in Fig 1. (TIF) [file pgen.1009626.s001.tif]

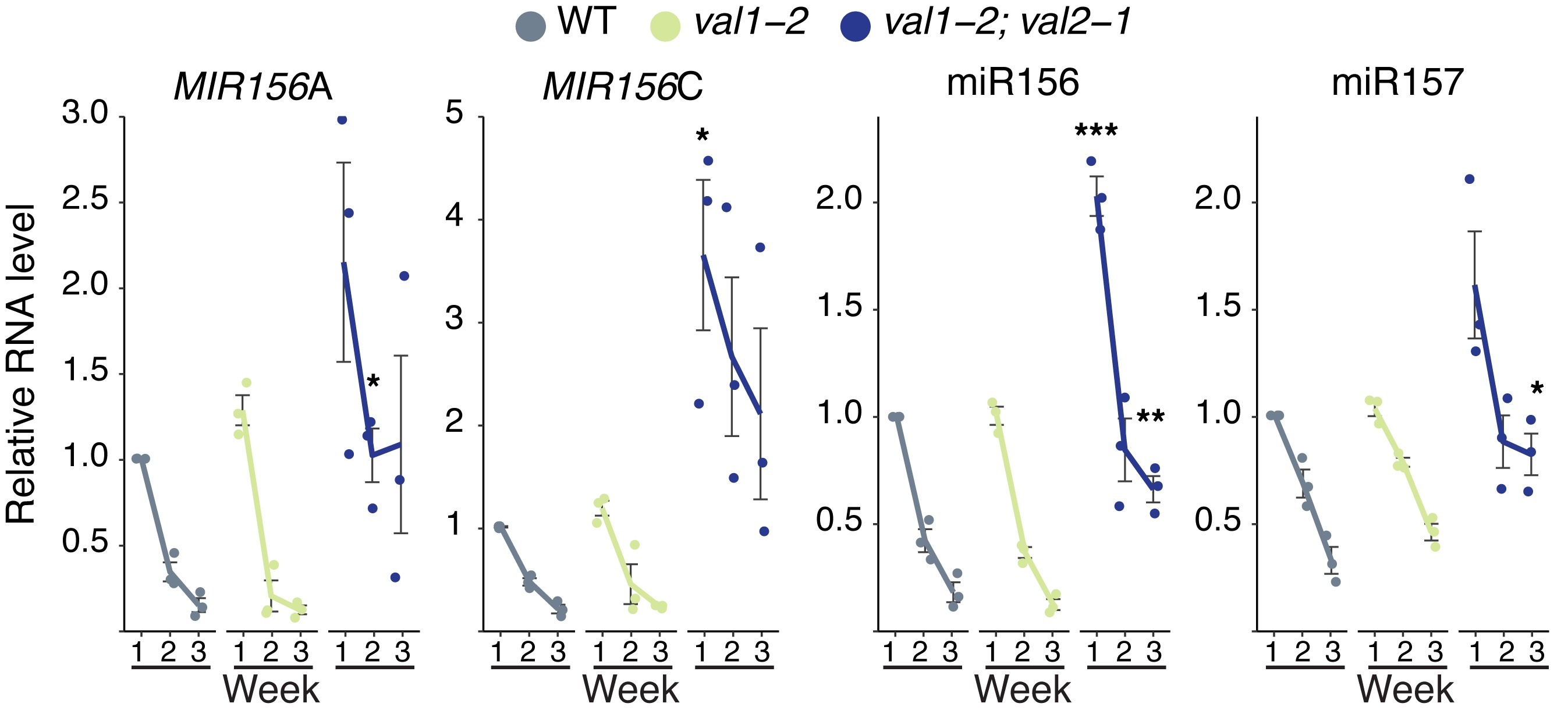

Supplement: S2 Fig — qRT-PCR analyses of gene expression in shoot apices with leaf primordia (LP) ≥ 1mm removed at 1, 2 and 3 weeks. All plants were grown in SD conditions. Each data point represents a biological replicate and is the average of three technical replicates. Coloured lines represent the mean and black lines mean±s.e.m. Asterisks represent significant differences between WT and val mutants at the same time point, calculated by an unpaired two-tailed t-test with a Bonferroni correction for multiple comparisons (* P < 0.025; ** P < 0.005; *** P < 0.0005). (TIF) [file pgen.1009626.s002.tif]

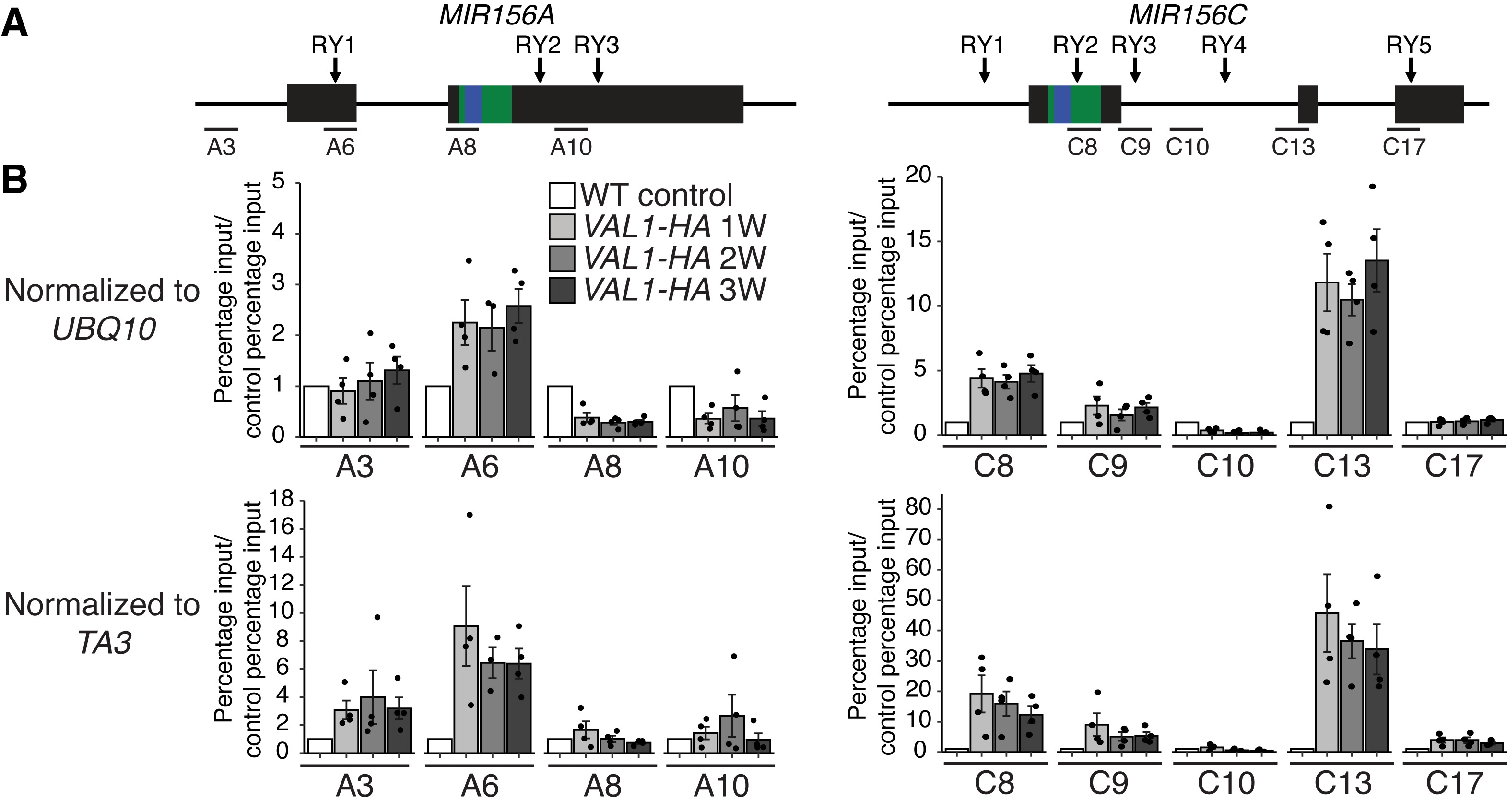

Supplement: S3 Fig — (A) Schematic depicting the location of primers used for ChIP-qPCR, the sequences encoding the miR156 hairpin and mature miRNA are coloured green and blue respectively. (B) Anti-HA ChIP-qPCR of WT Col control plants at 2W and VAL1::VAL1-HA; val1-2; FRI-Sf2 plants at 1, 2 and 3W of growth. The data is presented as percentage input normalized to a control locus (UBQ10 or TA3) and is displayed relative to WT. Each data point represents a biological replicate and is the average of three technical replicates, bars represent the mean and error bars the mean±s.e.m. Plants were grown in SD conditions. (TIF) [file pgen.1009626.s003.tif]

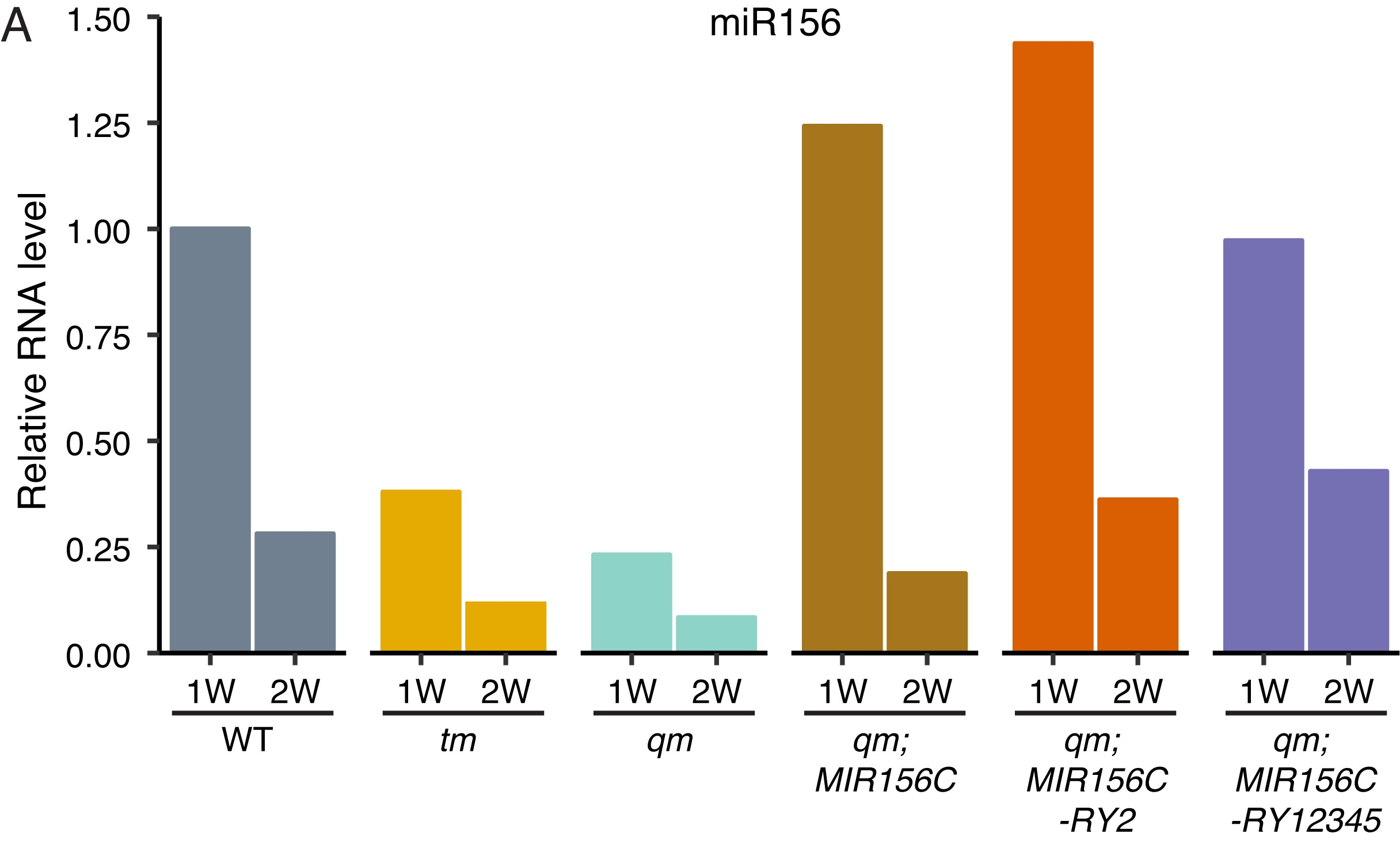

Supplement: S4 Fig — (A) qRT-PCR analyses of gene expression in shoot apices with LP ≥ 1mm removed at 1 and 2 weeks. Bars represent the average of three technical replicates for a single biological replicate of pooled T1 plants, at least 15 independent T1 plants were pooled for each sample. qm = mir156a mir156c mir157a mir157c quadruple mutant, tm = mir156a mir157a mir157c triple mutant. Plants were grown in SD conditions. (TIF) [file pgen.1009626.s004.tif]

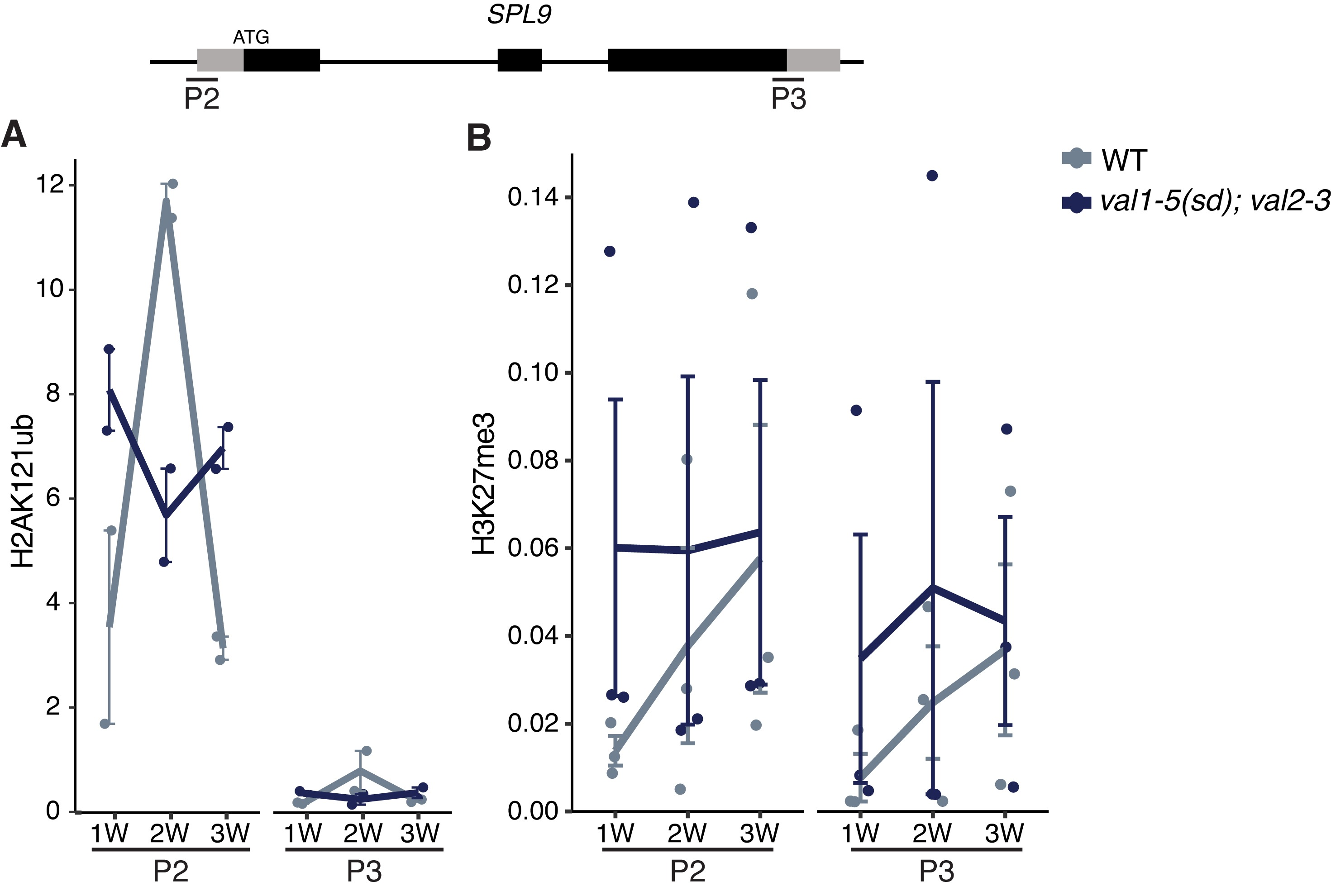

Supplement: S5 Fig — (A, B) Temporal analysis of histone modification scalculated by ChIP-qPCR. Each data point represents a biological replicate and is the average of three technical replicates. Lines represent the mean and bars represent the mean±s.e.m., (A) H2AK121ub values are relative to input and normalised to ABI3 as an internal control. (B) H3K27me3 values are relative to H3 and normalised to STM as an internal control. Plants were grown in SD conditions. (TIF) [file pgen.1009626.s005.tif]

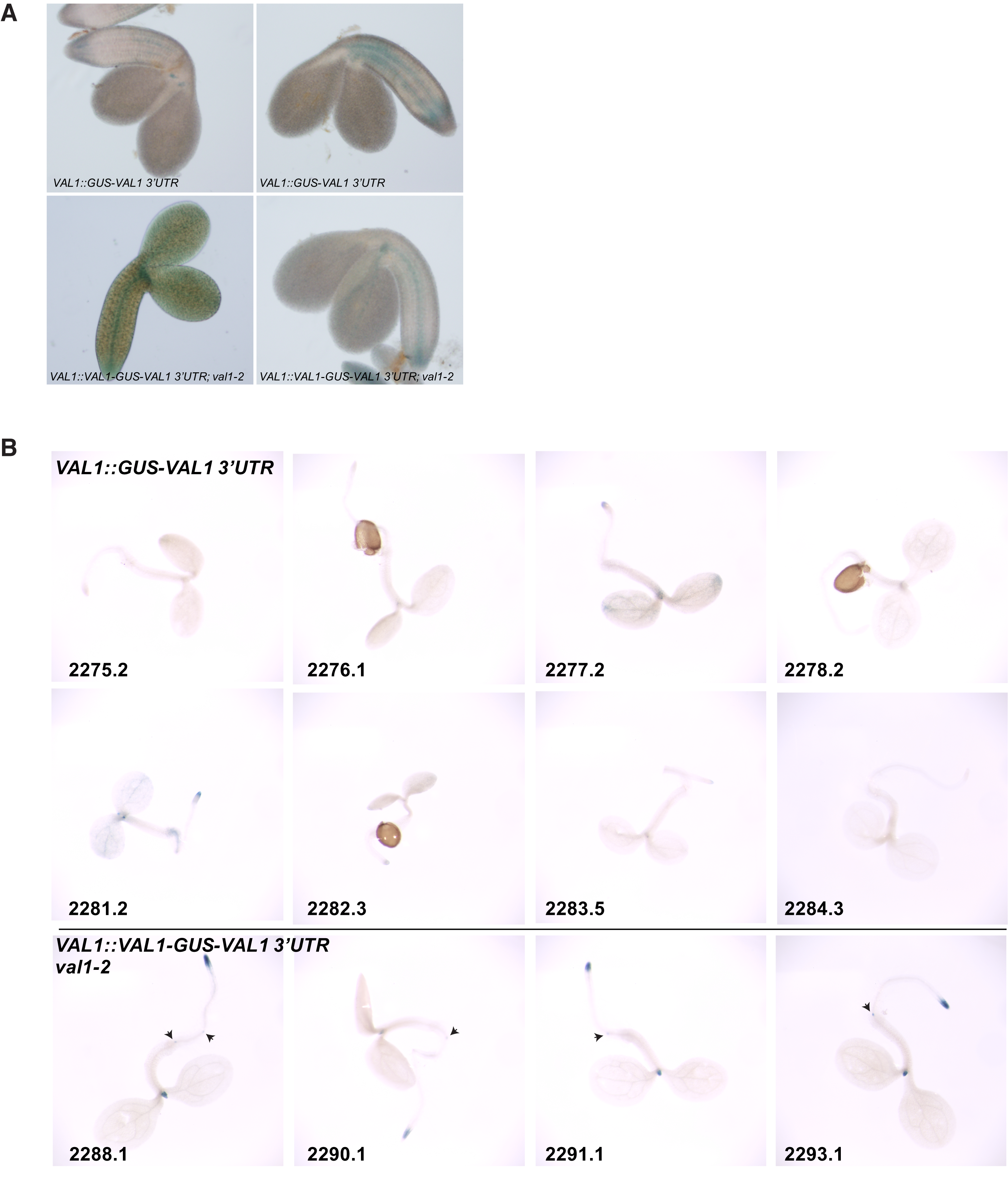

Supplement: S6 Fig — (A) Torpedo-stage embryos of two-independent homozygous transgenic lines each expressing a transcriptional (top panel) or translational (bottom panel) VAL1-GUS reporter construct. (B) Seedlings at 3 DAG. Each number designates an independent homozygous transgenic line. Arrow heads point to initiating lateral root primordia. All plants were grown in LD. (TIF) [file pgen.1009626.s006.tif]

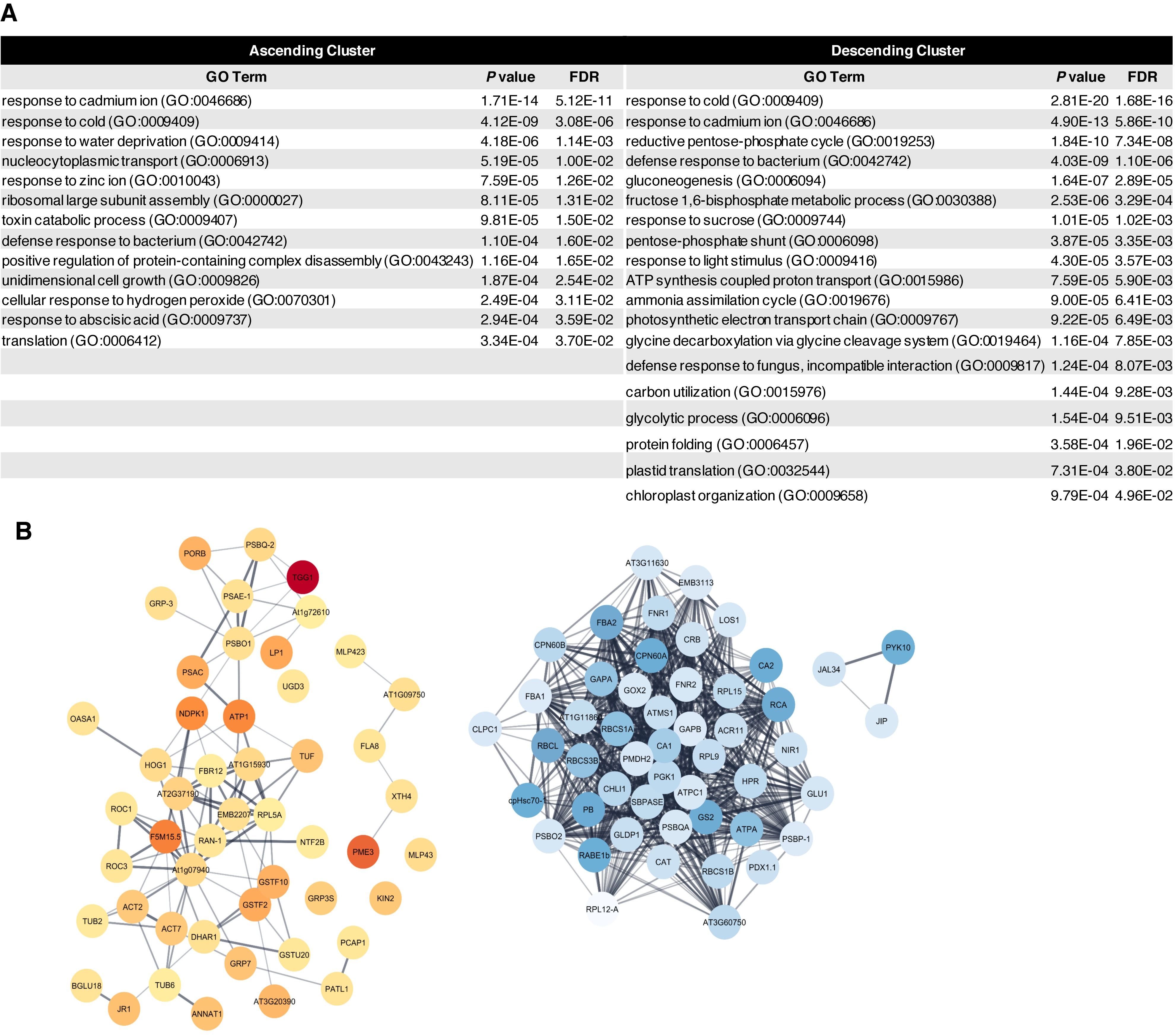

Supplement: S7 Fig — (A,B) The 50 proteins with the strongest increasing or decreasing trend score during vegetative development in the experimental sample only (see Methods for details). (A) GO terms enriched within the 50 proteins that have the strongest increasing or decreasing trend score. (B) Interaction networks for each set of 50 proteins, the darker the color the stronger the increasing (red) or decreasing (blue) trend score. (TIF) [file pgen.1009626.s007.tif]
