# Supplementary material for: VAL genes regulate vegetative phase change via miR156-dependent and independent mechanisms
Source: PLoS Genet. 2021 Jun 28;17(6):e1009626. doi: 10.1371/journal.pgen.1009626 (PMC8270478; doi:10.1371/journal.pgen.1009626)
Supplement: Attachment — Submitted filename: PLOS Gen response to reviewers.docx [file pgen.1009626.s012.docx]

We thank the reviewers for their careful reading of the manuscript and generous and useful comments. Our responses to their specific comments are detailed below.

**Reviewer 1**

*Materials and methods did not explain at well how miR156 and miR157 mature transcript levels were quantified. Probably this was by very standard methods, but they should be described in better detail.*

We have updated the text to refer to the ‘stem-loop’ nature of reverse-transcription primers for mature miRNAs and included references to the original methods papers.

*Lines 572 – 574 states that VAL1 VAL 2 repress SPL9 expression by miR156 independent means, however the results section showed the opposite e.g. expression of miR156 resistant SPL9 transgene is much higher in VAL1+ than in val1 mutants so VAL1 activity promotes SPL9 expression albeit likely indirectly.*

Thank you for pointing this out. We have corrected this in the text, and been more explicit about the interaction between VALs and SPL9 in the results section.

**Reviewer 2**

1. *Line 157 – val2-4 is a newly described val2 allele – has expression of the locus been tested to determine the effect of the T-DNA insertion?*

We tested accumulation of *VAL2* transcripts in *val2-4* and found a significant, but not complete, reduction. Although given the T-DNA is located within a *VAL2* exon the functionality of the residual transcripts is unclear. We have updated the text and Fig S1 with this expression data.

*2. Fig 1E (line 163), Fig.4D – The blade base angle results are not intuitive. For WT, the expectation would be an increase of blade base angle in adult leaves. In contrast, the angle decreases in leaf 5 compared to leaf 3. Can the authors indicate how the blade angle is defined in this particular quantification?*

We measure the ‘blade base angle’ as the angle of the blade where it joins the petiole (effectively two tangents of the blade base that intersect at the petiole). Therefore a decrease in blade angle is expected during development as the base of the leaf transitions from being almost flat to V-shaped. This is best indicated by the leaf silhouettes in Fig. 5B. We have added a Methods section on leaf measurements to clarify this.

*3. Fig1C – the color-coding should be changed as the shades of blue and green are difficult to be differentiated in pdf or print*

We have changed the colour scheme to make the genotypes more distinguishable.

*4. Lines 164-166: A comparison is made between val1-5(sd); val 2-3 and val1-2;val2-3, where the latter is not shown (analysed) and a reference to previous works (in which the mutants are grown in vitro on MS medium) is made. I would argue that the difference may be caused by different growth conditions and if such comparison should be made, the mutants should be tested in parallel. I therefore think that the association between the val1-5(sd) semi-dominance and VAL2 interaction is not sufficiently justified.*

We agree that the growth conditions may contribute to differences in phenotypic strength, and a comparison of val1-5(sd); val 2-3 and val1-2; val2-3 both grown on MS would be insightful. However, despite multiple attempts, we were not able to generate our own val1-2; val2-3 line on soil, whereas val1-5(sd); val2-3 has a relatively high germination rate on soil. Importantly, loss-of-function val2 alleles have a stronger effect in the val1-2 than val1-5(sd) background (Fig 1). Taken together we believe this provides reasonable evidence that val1-5(sd) is interfering with VAL2 activity. We have amended the text to include the latter line of evidence.

*5. Lines 203-204 (Fig 2A,B, S2 Fig) While I agree with the increase of miR156 in val mutants, I don´t think evidence supports the general conclusion that miR157 is increased upon loss of VAL activity. Increase is robustly visible in val1-2; val2-1 (Fig S2) but not in the other mutant/allelic combinations. In fact, there is significantly less miR157 in val1-5(sd); val 2-3 in youngest leaf primordia.*

We agree that our statement on miR157 expression was too vague and inconsistent with the young leaf primordia data. We have modified the text to state that “Loss of *VAL* activity also produced a slight increase in miR157 levels at later stages of development”

*6. Line 229: formulation: “…we examined PRC2 activity in the val1-5(sd); val2-3…” PRC2 activity was not examined (and most likely it would be comparable if “only” recruitment is impaired). Please reformulate.*

We have altered “PRC2 activity” to “H3K27me3 accumulation”.

*7. ChIP-qPCR – the rationale behind the difference in quantification is unclear – Fig3B – H3K27me3 related to H3 and normalized to STM (H3K27me3 target) and Fig. 3F – H2Aub related to input and normalized to ACT7 (H3K27me3/H2Aub non-target). The stability of STM and ACT7 in terms of the modification occupancy is not shown.*

For consistency we have normalized our H2Aub deposition data to the H2Aub target ABI3. This has led to a change in our interpretation of the H2Aub data. Specifically, loss of *VAL* activity appears to have less of an effect on H2Aub deposition when normalized in this way. We have updated the results and discussion sections to reflect this

*8. Line764, line 510 – 512: VAL1-HA coIP experiment- the age of plants at harvest should be indicated in methods as well as in the main text/legend.*

We have updated the methods section to include details of the experimental time course.
